# A new surgical strategy for the treatment of tibial pilon fractures with MIPO facilitated by double reverse traction repositor

**DOI:** 10.1038/s41598-022-11150-7

**Published:** 2022-04-30

**Authors:** Bo Wang, Kuo Zhao, Zhucheng Jin, Junzhe Zhang, Wei Chen, Zhiyong Hou, Yingze Zhang

**Affiliations:** 1 Department of Orthopaedic Surgery, Third Hospital of Hebei Medical University, Hebei Province, No. 139 Ziqiang Road, Shijiazhuang, 050051 People’s Republic of China; 2 Key Laboratory of Biomechanics of Hebei Province, Shijiazhuang, 050051 Hebei People’s Republic of China; 3 Orthopaedic Research Institution of Hebei Province, Shijiazhuang, 050051 Hebei People’s Republic of China; 4 NHC Key Laboratory of Intelligent Orthopaedic Equipment, The Third Hospital of Hebei Medical University, Shijiazhuang, People’s Republic of China; 5 Chinese Academy of Engineering, Beijing, 10088 People’s Republic of China

**Keywords:** Diseases, Medical research

## Abstract

The present study aims to introduce a technique combining double reverse traction repositor (DRTR) with minimally invasive plate osteosynthesis (MIPO) in the surgical treatment of pilon fractures and to observe the efficiency of this approach during a short-term follow-up period. From January to December 2018, patients with pilon fractures who were treated by MIPO with DRTR were reviewed. The demographic and fracture characteristics, surgical data, and prognostic data of 24 patients were extracted. In all 24 patients, closed reduction was achieved with the MIPO technique, and excellent functional and radiological outcomes were observed. The average duration of surgery and intraoperative blood loss were 95.0 ± 14.2 min and 152.1 ± 52.1 ml, respectively. A mean of 16.0 ± 1.9 intraoperative fluoroscopies were conducted. At the 12-month follow-up evaluation, the average AOFAS score was 85.2 ± 5.1. Anatomic or good reduction was observed in 23 (95.8%) patients. The mean ranges of motion of dorsiflexion and plantarflexion were 11.0 ± 2.7 and 32.7 ± 11.1, respectively. Two patients with deep venous thrombosis and one patient with wound non-purulent exudate were noted. Additionally, the wounds healed after routine dressing change. No other complications, including skin necrosis or delayed, non-union or malunion, were observed. The new strategy combining DRTR with MIPO in the treatment of pilon fractures allowed excellent radiological and clinical outcomes and a low postoperative complication rate to be achieved in a short-term follow-up period. Further large sample and comparative studies should be conducted to validate our results.

## Introduction

Tibial pilon fractures are uncommon conditions involving osteoarticular, metaphyseal, and soft-tissue compromise and account for 5–10% of tibial fractures and 1.03% of all adult fractures^1,2^. They were first proposed by Destot et al. in 1911 and were renamed tibial plafond fractures by Bonin in 1950. For complex fracture characteristics, such as less muscle cover and poor vascularity, their management presents orthopaedic surgeons with significant challenges. The mechanism of injuries includes high-energy trauma resulting from an axial load and low-energy trauma with a rotational injury. Increased articular comminution, severe soft-tissue injury, large osseous deficits and articular cartilage damage are common in pilon fractures caused by high-energy trauma. Proper management of metadiaphyseal osseous deficits and circumferentially compromised soft tissue is required to achieve favourable outcomes.

Historically, nonoperative treatment was the preferred method for pilon fractures due to their nonreconstructable characteristics and poor functional outcomes. In 1969, Rüedi and Allgöwer reported the application of open reduction internal fixation (ORIF) in the treatment of pilon fractures and found improved clinical outcomes in patients^3^. To date, various methods have been demonstrated in the management of pilon fractures, including ORIF, minimally invasive plate osteosynthesis (MIPO), external fixation (EF) and primary arthrodesis (PA)^4,5^. ORIF is a common choice for tibial pilon fractures and can help restore anatomic structures of the articular surface. Extensive dissection of the periosteum and soft tissue has been suggested to be associated with a high rate of infection, wound healing complications, delayed union and nonunion^6,7^. EF is usually regarded as a temporary option for patients with open fractures or serious soft tissue injury. It is rarely regarded as a definitive treatment for causing malunion, infections, and ankle stiffness^8^. Some surgeons suggest choosing PA for nonreconstructable pilon fractures^9,10^. While acceptable outcomes could be obtained, they only represent a strict option for selecting patients.

MIPO is a suitable substitute for the surgical strategy of pilon fractures, which aims to minimize soft tissue dissection by indirect reduction and stable fixation^11,12^. Previous studies have reported satisfactory outcomes and fewer wound healing complications in patients with MIPO^12,13^. Although the blood supply of soft tissue and periosteum can be preserved commendably, MIPO makes little contribution to the reduction of fractures, especially for compression fractures involving the articular surface. Therefore, malreduction, angular deformities, and traumatic arthritis are not uncommon postoperatively^14^.

As a traction device, the double reverse traction repositor (DRTR) is always applied to help reduce lower limb fractures, including intertrochanteric fractures, femoral shaft fractures, distal femur fractures and tibial plateau fractures^15–18^.

To help achieve a closed reduction in MIPO, the authors introduced the application of DRTR in the treatment of pilon fractures. The present study aims to introduce DRTR combined with MIPO in the surgical treatment of pilon fractures and to observe its efficiency in short-term follow-up.

## Patients and methods

This study was approved by the ethical committee of Hebei Medical University Third Affiliated Hospital, and all patients provided informed consent before surgery. This study was performed in accordance with the guidelines of the Declaration of Helsinki. From January to December 2018, patients with pilon fractures who were treated by MIPO with DRTR in our department were reviewed. According to the inclusion and exclusion criteria, a total of 24 patients were included in this study. The inclusion criteria were patients with AO/OTA type 43 B and 43 C pilon fractures, adult patients (aged at least 18 years), surgical treatment by MIPO with the help of DRTR, and followed for 1 year or more. The exclusion criteria were as follows: (a) multiple fractures, open fractures or pathologic fractures; (b) accompanying neural or vascular damage; (c) old fracture (time from injury to surgery > 21 days); (d) episodes of compartment syndrome; (f) patients with incomplete data.

The demographic and fracture characteristics are shown in Table 1. Among all 24 patients, 20 were males. The average age was 44.6 ± 10.3 years (range from 20 to 63 years). The average body mass index (BMI) was 24.8 ± 3.3 kg/m^2^. Eight patients had left-side fractures, and none of the patients had bilateral fractures. The classification of fractures was performed with the AO/OAT classification system (AO/OAT 43-B and 43-C). Overall, there were 13 AO/OAT 43-B patients and 11 AO/OAT 43-C patients. Associated fibula fractures were present in 14 (58.3%) patients. Eleven (78.6%) of them were receiving fibula osteosynthesis. Preoperative examinations were carried out actively prior to surgery.

**Table 1:** Patient demographic data and fracture characteristics.

Variables	Number
Total patient, no. (%)	26 (100.0)
Age (years), mean ± SD	44.6 ± 10.3
Gender (male), no. (%)	20 (83.3)
Side (left), no. (%)	8 (33.3)
**AO Classification, no. (%)**	
43 B	13 (54.2)
43 C	11 (45.8)
Injury mechanism (high energy), no. (%)	10 (41.7)
BMI (kg/m^2^), mean ± SD	24.8 ± 3.3
Fibula fractures, no. (%)	14 (58.3)

Abbreviation: *SD* standard deviation.

### Surgical technique

All operations were performed by the same team, including 1 chief physician and 2 attending physicians. The operations were conducted on a radiolucent operating table with the patients in a supine position. Routine skin preparation and draping were carried out after anaesthesia, and the tourniquet was set as 280 mmHg. According to preoperative evaluations, skin preparation of the contralateral anterior superior iliac spine area was conducted for autologous iliac crest grafts in patients with severe comminuted fractures and serious bone defects.

#### Step 1 The reduction of fractures under DRTR

**Figure 1:**
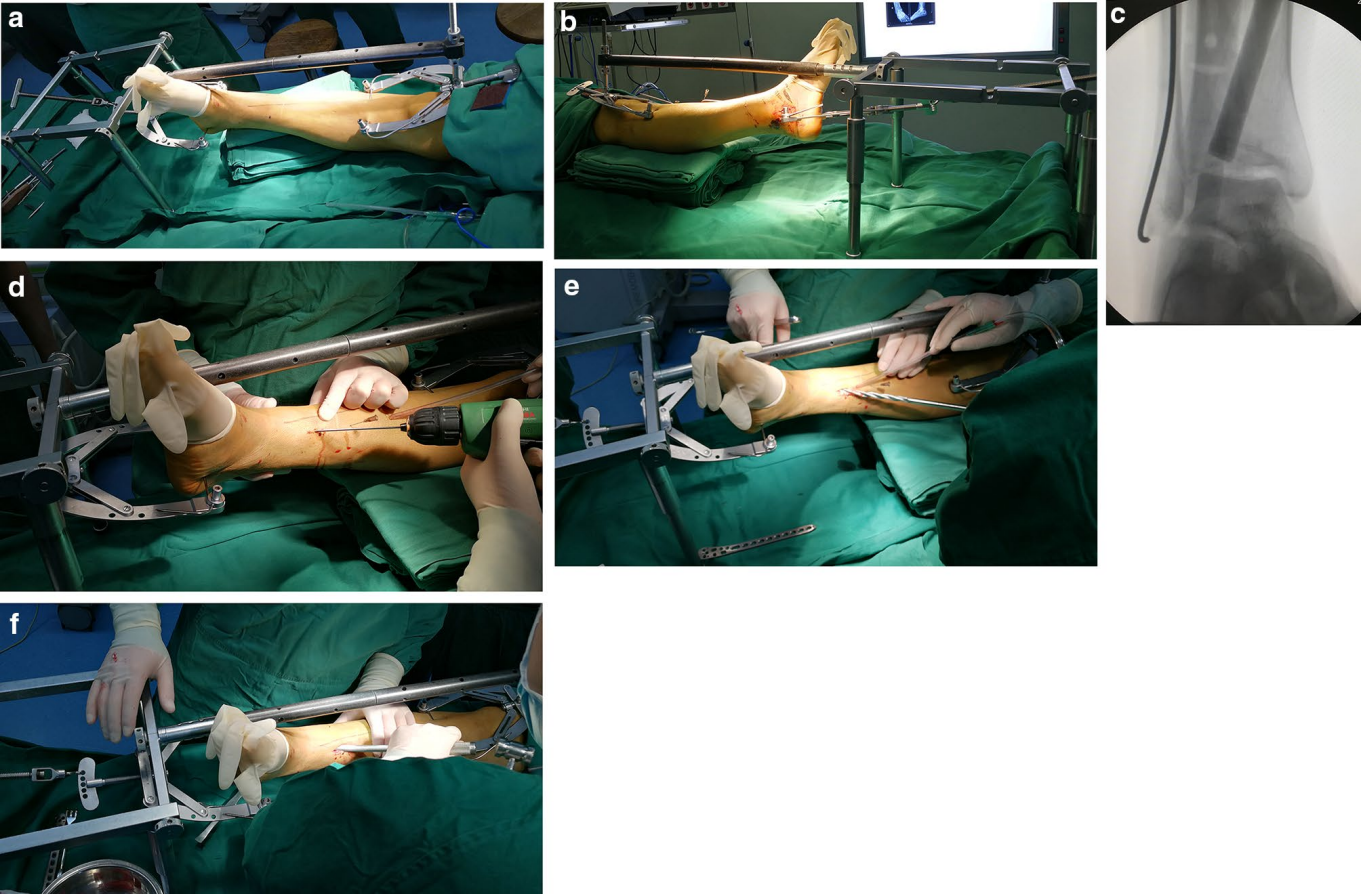
The process of fractures reduction with DRTR. (**a**) and (**b**) The anterior–posterior view and lateral view of DRTR in the surgery. (**c**) The reduction of collapsed fractures under fluoroscopy. (**d**) A Kirschner wire was inserted to determine the position of the collapsed fractures. (**e**) A series of pulp chamber burs were used to make a bone channel. (**f**) Striking the bone tamp to reduce the collapsed fractures.

First, the DRTR was installed (Fig. 1a and b). The DRTR was mainly composed of a foldable reduction bracket, carbon fiber connecting rod and two traction bows. A 2.5 mm diameter Kirschner wire was inserted into the middle and upper thirds of the tibia and calcaneal body, respectively, and a traction bow was connected. The proximal traction bow was connected to the reduction bracket through the carbon fiber connecting rod, and the distal traction bow was directly connected to the rotary handle of the reduction bracket. After the installation of the DRTR, the traction force between the two traction bows could be generated by rotating the handle counter-clockwise. With the rotation of the handle, the traction force increased, and the surrounding skin, ligament and capsule around the ankle joint were stretched and tensed. The forces from the ligaments, capsule and skin around the ankle joint could help reset most of the displaced fractures. If there were residual fractures with separation or angular displacement, manual reduction or additional incision was conducted to reduce the fractures.

With regard to the compression fractures that could not be reduced by traction, the reduction was completed by a special bone tamp (Fig. 1c). A 2.5 mm diameter Kirschner wire was inserted towards the collapsed fracture fragment at the juncture 6–8 cm above the medial malleolus and the anterior and middle third of the medial tibia under fluoroscopy (Fig. 1d). The insertion was stopped at approximately 1 cm from the collapsed fracture fragment. Pulp chamber burs were used to make a channel along with the Kirschner wire, in which the size of pulp chamber burs was from small to large until a maximum size of 14 mm (Fig. 1e). Then, the reduction of collapse fractures was completed with a series of strikes of bone tamp through the channel under fluoroscopy (Fig. 1f).

#### Step 2 Autologous iliac crest graft

**Figure 2:**
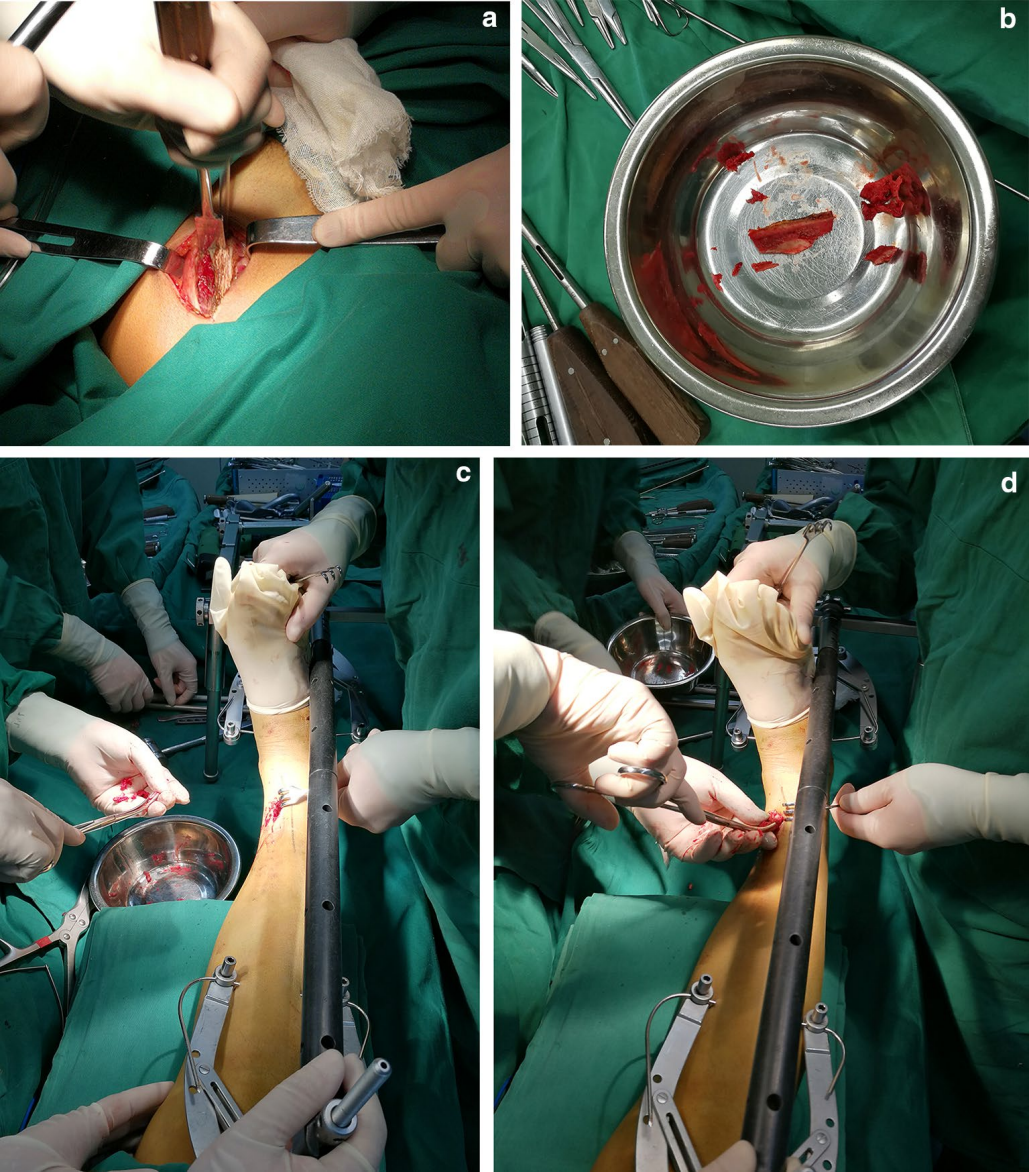
The key messages of autologous iliac crest graft. (**a**) The autologous bone graft was obtained from the contralateral iliac bone. (**b**) The superior and lateral cortex were preserved in the transplanted bone. (**c**) The cancellous bone fragments was implanted. (**d**) the suitable length and diameter of bicortical iliac bone was implanted.

For patients with severe comminuted fractures and serious bone defects, an autologous iliac crest graft was used. The autologous bone graft was obtained from the contralateral iliac bone (Fig. 2a). The superior and lateral cortices were preserved in the transplanted bone, which could provide strong support for the collapsed fragments (Fig. 2b). During bone grafting, a certain amount of cancellous bone fragments was implanted first (Fig. 2c). Then, a suitable length and diameter of bicortical iliac bone was implanted (Fig. 2d).

#### Step 3 Fixation with the MIPO technique

**Figure 3:**
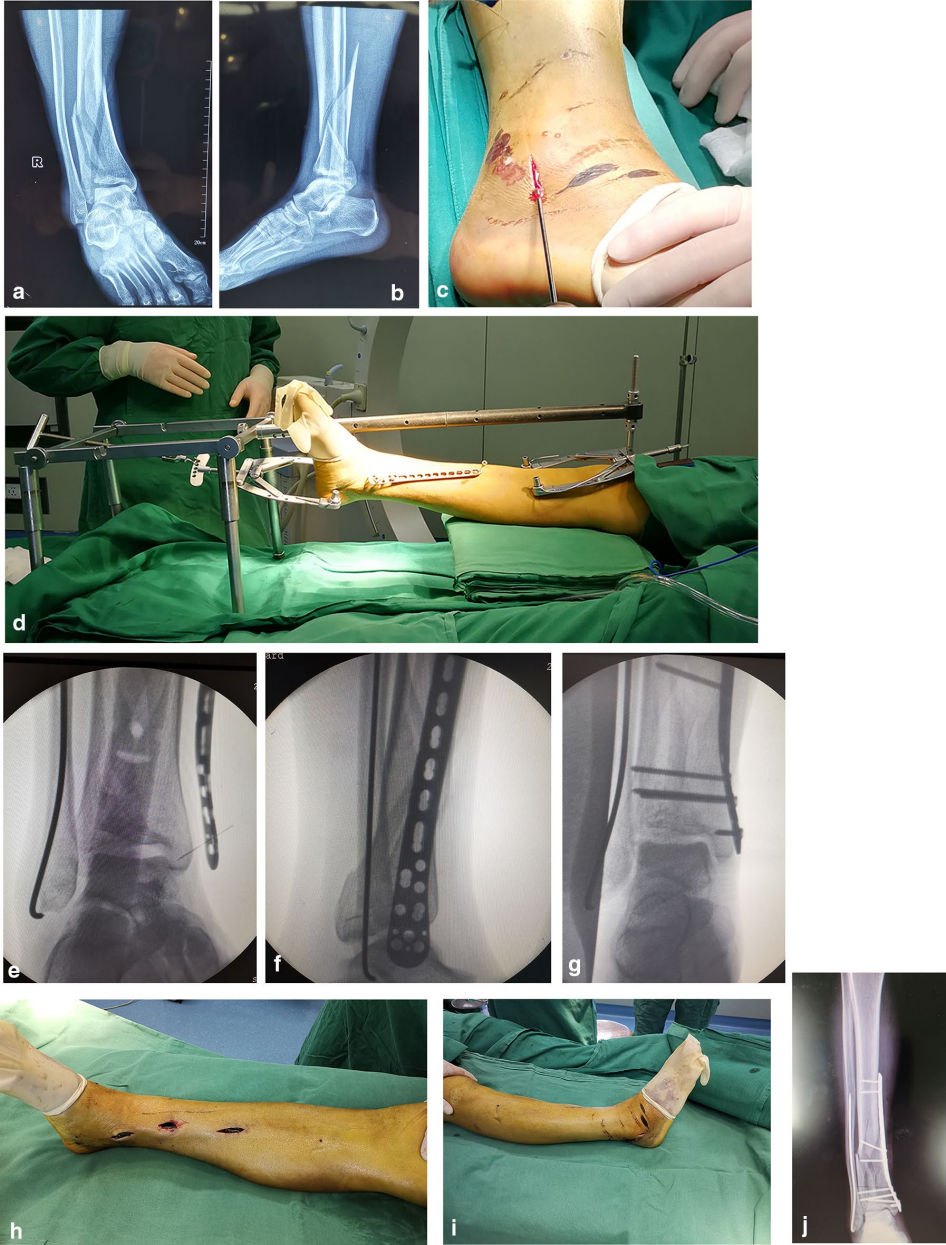
Fixation with MIPO technique. (**a**) and (**b**) The anteroposterior and lateral X-rays of a pilon fractures. (**c**) The fibular was fixation firstly by a 2.5 mm diameter Kirschner wire. (**d**) The syringe needles were used to locate the position of the plate to achieve minimally invasive incision. (**e**), (**f**) The plate was adjusted to the best position under fluoroscopy. (**g**) Intraoperative fluoroscopy showed that excellent fracture reduction was obtained. The surgical incision was shown in (**h**) and (**i**). (**j**) The plate was fixation with MIPO technique.

After satisfactory reduction of fractures was observed, an anatomically configured locking compression plate was fixed with the MIPO technique (Fig. 3a–j).

### Postoperative management and data collection

Radiological evaluation of the fracture was performed after surgery. Standard antibiotic prophylaxis and conventional low molecular weight heparin sodium were applied postoperatively. Patients were encouraged to perform tolerable range active and passive motion and muscle strengthening exercises on the day after the operation. Partial weight-bearing was permitted at the 8th week after surgery. The time of full weight-bearing was dependent on the healing of fracture according to the radiographic evaluation.

Surgical data, including the duration of surgery, blood loss, open reduction or closed reduction, were reviewed. Meanwhile, postoperative complications, including wound infection, cutaneous necrosis, deep venous thrombosis, delayed union, non-union, malunion, and fixation failure, were extracted. The patients were followed up at 1, 3, 6, and 12 months postoperatively and then every half year. The radiographic and clinical outcomes were collected. Radiographic evaluation was conducted by one radiologist. According to Burwell and Charnley et al.^19^ the reduction of fracture was classified into anatomic, good, and poor. Clinical evaluations consisted of range of motion (ROM), American Orthopaedic Foot & Ankle Society (AOFAS) scale and visual analogue scale (VAS)^20,21^. The AOFAS scale consist of nine items including three subscales (pain, function and alignment). The maximal score of pain is 40 points, which indicate no pain. Function involves 7 items, and a score of 50 points indicate full function. Alignment involves 1 item, and a maximal score of 10 points indicate good alignment. The AOFAS results were separated into excellent (> 90 points), good (80–90 points), fair (70–79 points) and poor (< 70 points). VAS is divided into 10 points with a smallest score of 0 point indicating no pain and a maximal score of 10 points for severe pain, and the middle part for different degrees of pain. And the ROM was evaluated during post-operative.

### Ethics approval and consent to participate

Permission was obtained from the Hebei Medical University Third Affiliated Hospital Ethics Board, and informed consent was obtained from all patients in this study.

## Results

### Intraoperative outcomes

The intraoperative outcomes are shown in Table 2. Ten surgeries were conducted under general anaesthesia. The average duration of surgery was 95.0 ± 14.2 min. The mean intraoperative blood loss was 152.1 ± 52.1 ml. A mean of 16.0 ± 1.9 intraoperative fluoroscopies was shown. Among the 14 patients with fibula fractures, 11 (78.6%) received fibula osteosynthesis. All surgeries were successfully conducted by MIPO, and open reduction was not observed in any of them.

**Table 2:** Details of surgical data.

Variables	Number
Duration of surgery (min), mean ± SD	95.0 ± 14.2
Intraoperative blood loss (ml), mean ± SD	152.1 ± 52.1
Intraoperative fluoroscopy times, mean ± SD	16.0 ± 1.9
Anesthesia(general), no. (%)	10 (41.7)
Open reduction, no. (%)	0 (0.0)
Fibula osteosynthesis	11 (78.6)

Abbreviation: *SD* standard deviation.

### Clinical and radiological results

**Figure 4:**
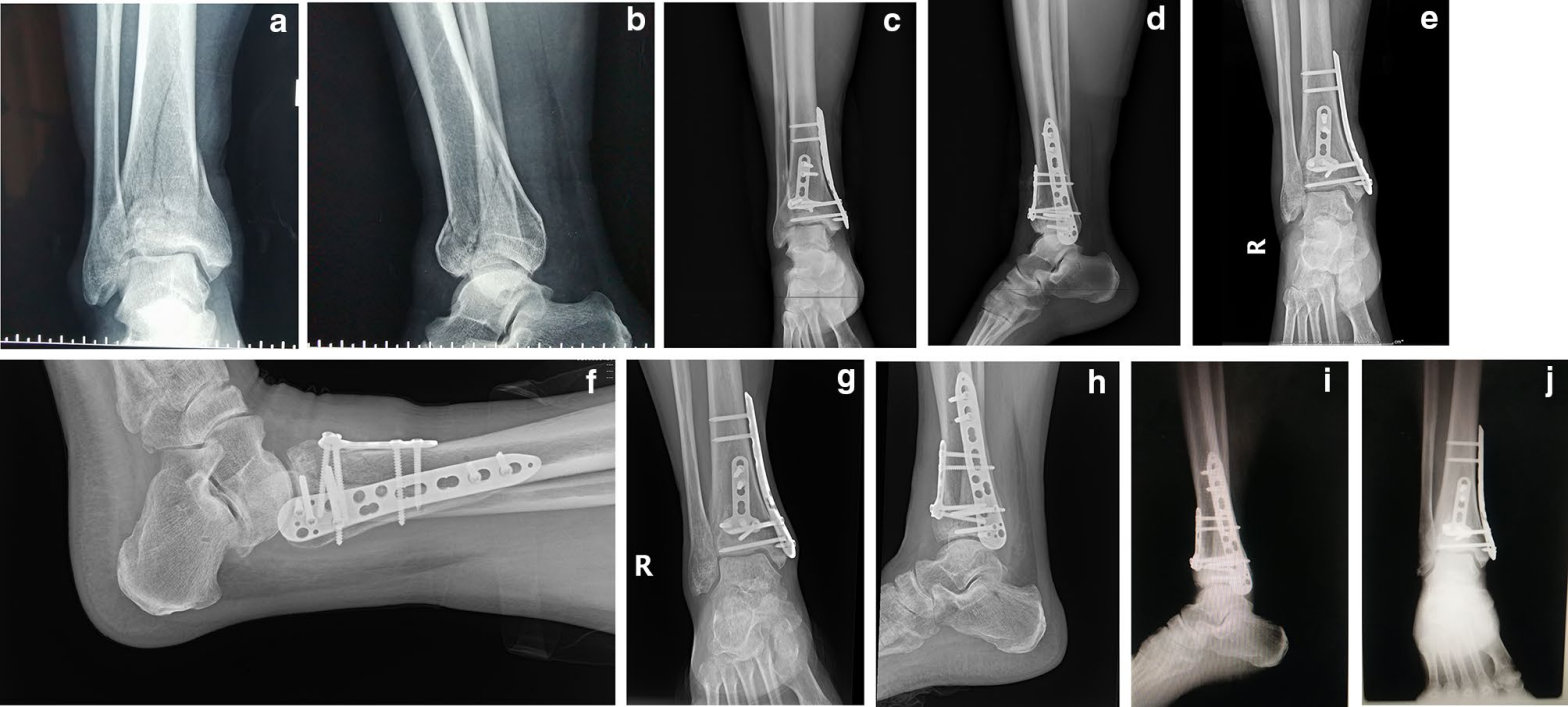
The anteroposterior and lateral X-rays of a pilon fractures. (**a**) and (**b**) Preoperative anteroposterior and lateral X-rays. (**c**) and (**d**) Anteroposterior and lateral X-rays on the day after surgery. (**e**) and (**f**) Anteroposterior and lateral X-rays 1 month after surgery. (**g**) and (**h**) Anteroposterior and lateral X-rays 3 month after surgery. (**i**) and (**j**) Anteroposterior and lateral X-rays 12 month after surgery.

The clinical and radiological results at the 12-month follow-up are shown in Table 3. All fractures healed within 6 months, of which 75% were observed at 3-month follow-up (Fig. 4a–j). The average fracture healing time was 3.7 months. The average AOFAS score was 85.2 ± 5.1. According to Burwell-Charnley, anatomic or good reduction was observed in 23 (95.8%) of patients. An average VAS score of 1.3 was obtained. Meanwhile, the mean ROM of dorsiflexion and plantarflexion were 11.0 ± 2.7 and 32.7 ± 11.1, respectively. In terms of postoperative complications, 2 deep venous thromboses were observed. The thromboses were recanalized after receiving therapeutic doses of low-molecular-weight heparin sodium (4250 IU every 12 h). One patient with wound non-purulent exudate was observed, and the wound healed after routine dressing change. No skin necrosis was observed. No other complications were observed.

**Table 3:** Prognostic comparison.

Variables	Number
Follow-up time (months), mean (range)	14.3 (12–18)
AOFAS scores, mean ± SD	85.2 ± 5.1
**Burwell-Charnley, no. (%)**	
Anatomic reduction	14 (58.3)
Good reduction	9 (37.5)
Poor reduction	1 (4.2)
VAS scores, mean (range)	1.3 (0–3)
Dorsiflexion (°), mean ± SD	11.0 ± 2.7
Plantarflexion (°), mean ± SD	32.7 ± 11.1
**Complications, no. (%)**	
Deep venous thrombosis	2(8.3)
Wound infection	1(4.2)
Skin necrosis	0(0.0)
Delayed, non- or mal- union	0(0.0)
Traumatic osteoarthritis	1(4.2)

Abbreviation: *SD* standard deviation.

## Discussion

This study was conducted to observe the functional outcomes, radiological outcomes and complications of tibial pilon fractures treated with DRTR combined with MIPO. The results exhibited successful closed reduction with the MIPO technique, excellent functional and radiological outcomes, and a low postoperative complication rate in the short-term follow-up period.

The MIPO technique is an ideal substitute for the surgical treatment of pilon fractures with respecting soft tissue and blood supply. Kim et al.^22^ reviewed 28 pilon fracture patients in their hospital whose operations were conducted through the MIPO technique. They found that all patients presented good ROM and low wound complication rates in their study. Barış et al.^23^ detected the long-term outcomes of pilon fractures treated with the MIPO technique. Their study showed that MIPO was a reliable technique that provided satisfactory clinical outcomes and a high fracture healing rate. In a recent study, Wu et al.^24^ reported the combination of the MIPO technique with an anterior curved incision for pilon fractures. The results showed good functional recovery and a low complication rate. In addition, the MIPO technique could also be used as an alternative in the primary arthrodesis of pilon fractures. Carlo et al. proposed an innovative MIPO technique for primary arthrodesis in the treatment of pilon fractures, and satisfactory results were observed^25^. However, disadvantages including potential malreduction or angular deformities, playing little role in helping the reduction of fractures, limit the use of MIPO in the treatment of pilon fractures, especially compression fragments^14^.

Excellent reduction is essential for obtaining satisfactory bone union and good functional outcomes. To achieve good reduction, various attempts in the surgical treatment of pilon fractures with MIPO have been presented in previous studies, including novel surgical incisions and different surgical strategies^12,22,24^. However, no traction device has been reported to facilitate the treatment of pilon fracture with the MIPO technique. The DRTR is an efficient traction device that was designed to achieve the reduction of lower limb fractures^15,26^. A previous study described that the DRTR could help achieve the reduction of femur shaft fractures, according to Zhang et al.^27^ Zhao et al. reported that the DRTR approach could be used to facilitate the reduction of intertrochanteric fractures^15^. In a recent study, DRTR was applied to achieve the reduction of distal femur fractures by Lian et al.^28^ In our previous studies, the combination of the MIPO technique with DRTR was used in the treatment of bicondylar tibial plateau fractures^18^. The reduction of depression fragments was reduced by a cylindrical metal bone tamp through a bony tunnel. The radiographic and clinical results were excellent.

The present study demonstrated the successful combination of DRTR with the MIPO technique in the treatment of pilon fractures. According to the Burwell-Charnley score, 95.8% of patients were observed to have anatomic or good reduction in this study, which was consistent with the findings of previous studies^24^. Excellent ankle ROM was shown at the 12-month follow-up evaluation. The mean ROM was 11.0 ± 2.7° for dorsiflexion and 32.7 ± 11.1° for plantarflexion, which was similar to the results of Vidovic et al.^3,29^ One prominent advantage of the MIPO technique was the low wound infection or skin necrosis rate. Previous studies reported that the rate of wound infection following tibial pilon fracture surgeries with MIPO ranged from 4.7 to 18.1%^24,29^. One patient with wound nonpurulent exudate was noted, and the wound healed after routine dressing change. No other complications were found. The low postoperative complication rate might be associated with the small sample size of this study. All surgeries were completed through closed reduction in this study, which might be associated with the small sample size of this study. For irreducible fractures, open reduction will be seen as the final choice.

The precautions and technical points are summarized as follows. When used in the treatment of pilon fractures, the proximal and distal traction sites of the DRTR were selected at the middle and upper third of the tibia and calcaneus. The traction force belonged to skeletal traction, which was sufficiently powerful to reduce fractures, including tibial and fibular fractures, and could maintain the reduction until the completion of fixation. With the continuous increase in traction force, the skin, muscles, ligaments and ankle capsule around the ankle become tense, and the compression of the surrounding soft tissue can help reduce the displacement fragments and maintain the reduction. Moreover, the traction force direction was almost parallel to the alignment of the lower extremities, which could help resume the mechanical axis of the distal tibia and decrease the possibility of malreduction. For the concomitant fibula fracture, it could be treated with closed reduction and internal fixation by a 3.0 mm Kirschner wire before the reduction of pilon fractures. And it also could be treated with MIPO technique under the help of DRTR. The operation of pilon fractures combined with joint depression required elevation of the articular fragments by impacting them with a cylindrical metal tamp. Reduction was achieved following successive impulse strikes to the tamp to restore the fragments step by step. In addition, autologous iliac crest grafts were conducted for patients with serious bone defects or geriatric patients. The application of cancellous bone fragments was conducive to the healing of fractures, and the bicortical iliac bone would provide stronger support to decrease the loss of reduction and prevent the development of traumatic osteoarthritis. For patients with associated fibula fractures were present in 14 (58.3%) patients. Eleven (78.6%) of them were receiving fibula osteosynthesis.

### Limitations

The present study introduced a new surgical strategy for the treatment of pilon fractures with MIPO. Excellent short-term outcomes were observed in all patients, but there remained several limitations in this study. First, it was a retrospective, nonrandomized control trial and noncomparative study. However, we extracted enough radiological and clinical evaluation indicators, which could help us master the short-term outcomes of this technique. Second, the inclusion of 24 patients with pilon fractures resulted in a small sample size, and not all types of pilon fractures were extracted in this study. In the future, a prospective, large sample, randomized control trial and comparative study should be conducted to further observe the radiological and clinical outcomes and postoperative complications of this method in the treatment of tibial pilon fractures with MIPO.

## Conclusion

The new strategy combining DRTR with MIPO in the treatment of pilon fractures resulted in excellent radiological and clinical outcomes and a low postoperative complication rate in the short-term follow-up period. Further large-sample and comparative studies should be conducted to validate our results.

## Data Availability

The datasets used and/or analysed during the current study are available from the corresponding author upon reasonable request.

## Abbreviations

MIPO: Minimally invasive plate osteosynthesis
DRTR: Double reverse traction repository
ORIF: Open reduction internal fixation
EF: External fixation
PA: Primary arthrodesis
BMI: Body mass index
AOFAS: American Orthopaedic Foot and Ankle Society
VAS: Visual analogue scale
ROM: Range of motion
